# Detecting Recovery Problems Just in Time: Application of Automated Linguistic Analysis and Supervised Machine Learning to an Online Substance Abuse Forum

**DOI:** 10.2196/10136

**Published:** 2018-06-12

**Authors:** Rachel Kornfield, Prathusha K Sarma, Dhavan V Shah, Fiona McTavish, Gina Landucci, Klaren Pe-Romashko, David H Gustafson

**Affiliations:** ^1^ School of Journalism and Mass Communication University of Wisconsin-Madison Madison, WI United States; ^2^ Department of Electrical & Computer Engineering University of Wisconsin-Madison Madison, WI United States; ^3^ Center for Health Enhancement System Studies University of Wisconsin-Madison Madison, WI United States

**Keywords:** self-help groups, substance-related disorders, supervised machine learning, social support, health communication

## Abstract

**Background:**

Online discussion forums allow those in addiction recovery to seek help through text-based messages, including when facing triggers to drink or use drugs. Trained staff (or “moderators”) may participate within these forums to offer guidance and support when participants are struggling but must expend considerable effort to continually review new content. Demands on moderators limit the scalability of evidence-based digital health interventions.

**Objective:**

Automated identification of recovery problems could allow moderators to engage in more timely and efficient ways with participants who are struggling. This paper aimed to investigate whether computational linguistics and supervised machine learning can be applied to successfully flag, in real time, those discussion forum messages that moderators find most concerning.

**Methods:**

Training data came from a trial of a mobile phone-based health intervention for individuals in recovery from alcohol use disorder, with human coders labeling discussion forum messages according to whether or not authors mentioned problems in their recovery process. Linguistic features of these messages were extracted via several computational techniques: (1) a Bag-of-Words approach, (2) the dictionary-based Linguistic Inquiry and Word Count program, and (3) a hybrid approach combining the most important features from both Bag-of-Words and Linguistic Inquiry and Word Count. These features were applied within binary classifiers leveraging several methods of supervised machine learning: support vector machines, decision trees, and boosted decision trees. Classifiers were evaluated in data from a later deployment of the recovery support intervention.

**Results:**

To distinguish recovery problem disclosures, the Bag-of-Words approach relied on domain-specific language, including words explicitly linked to substance use and mental health (“drink,” “relapse,” “depression,” and so on), whereas the Linguistic Inquiry and Word Count approach relied on language characteristics such as tone, affect, insight, and presence of quantifiers and time references, as well as pronouns. A boosted decision tree classifier, utilizing features from both Bag-of-Words and Linguistic Inquiry and Word Count performed best in identifying problems disclosed within the discussion forum, achieving 88% sensitivity and 82% specificity in a separate cohort of patients in recovery.

**Conclusions:**

Differences in language use can distinguish messages disclosing recovery problems from other message types. Incorporating machine learning models based on language use allows real-time flagging of concerning content such that trained staff may engage more efficiently and focus their attention on time-sensitive issues.

## Introduction

### Background

Digital health interventions have proliferated in recent years [1], and evidence suggests they can improve management of mental health issues, including substance use disorders (SUDs) [2,3]. Once their design is established, digital interventions can be disseminated with less expense and effort than face-to-face ones [4]. Such scalability is crucial for addressing SUDs, as demand for treatment dramatically outstrips available services [5]. In addition, although SUDs are chronic and relapsing [6,7], the help conveyed through technologies is ongoing and accessible. One recent clinical trial demonstrated that, relative to a control group, individuals who accessed a mobile phone–based recovery system reported reduction in risky drinking days by more than half over a year [8].

Substantial human labor also supports many effective digital health interventions. Some evidence suggests that, relative to interventions that lack human guidance, those that combine computerized tools with human support and coaching can enhance engagement and improve effectiveness of interventions [9]. The need for human expertize extends to interventions featuring peer-to-peer communication. Digital peer-to-peer interventions have involved “moderators” in various ways, including spurring and guiding discussion; monitoring forums for problematic content; and, crucially, providing just-in-time support to patients who are struggling, including through escalating contact or recommending treatment [10,11]. Through just-in-time support, moderators contribute to efficiency of health services at a systems level, making additional attention and resources available to those who most need them, while maintaining less intensive support for those at a lower risk level. Yet, attending to changing needs of an online health community poses a considerable challenge as participants can produce a massive volume of text exchanges [12]. Demands on staff represent a key hurdle in scaling up digital health interventions [13]. In this paper, we describe how automated linguistic analysis of text-based exchanges, and supervised machine learning, may play a role in managing moderator workflow in a technology-based recovery support system.

Our approach builds on the power of language as a signal of mental health risk, with linguistic cues being increasingly discernable through computational methods. Over the past several decades, researchers have amassed an extensive body of literature showing the promise of language to reveal individuals’ psychological traits, thoughts, feelings, and likely behaviors [14], including in social media contexts [15]. As similar ideas can be conveyed in different ways, individuals’ risk profiles emerge not only from the explicit content of their communication (ie, what topics authors are talking or writing about) but also from the style of their language (ie, *how* authors say what they say). In this study, we investigate how recovery challenges may emerge both through the individual words that authors use within a discussion forum as well as through general psycholinguistic dimensions of their messages (eg, affect, cognitive mechanisms), as captured through a dictionary-based approach. Leveraging these linguistic features, our goal is to find classifiers that can accurately label messages as conveying or not conveying recovery problems, allowing us to prioritize this content for review and intervention.

We consider several computational linguistic and machine learning approaches. First, we extract linguistic features of messages using 3 techniques: (1) a Bag-of-Words (BoW) approach representing each message in terms of word occurrences, (2) the Linguistic Inquiry and Word Count (LIWC) program (Pennebaker Conglomerates, Austin, TX) [16], which computes rates of language use within validated dictionaries corresponding to psychological and linguistic concepts, and (3) a novel hybrid approach combining important features from BoW and LIWC. We propose that BoW and LIWC have complementary strengths, with BoW attending to important words specific to the dataset (eg, those related to substance use), whereas LIWC attends to relevant psychological states (eg, anxiety, self-focus). We expect that a hybrid approach, capitalizing on the strengths of each, should outperform either LIWC or BoW. We test these techniques in the context of supervised machine learning models that have been utilized in social media contexts: support vector machines (SVM), decision trees, and boosted decision trees.

In interpreting performance of our computational linguistic and machine learning approaches, we consider some particularities of the domain of addiction recovery support, namely: (1) a low tolerance for false negatives, (2) a preference for understandability of the method to stakeholders, and (3) efficiency in processing language and classifying messages in real-time. In other words, in addition to considering overall accuracy of each classifier, we ask: Does it miss too many worrisome messages to be useful to forum moderators? Does it have face validity to a team of health professionals? And can we successfully implement it in real-time? To establish the utility and robustness of our approach, we test our classifiers in a separate iteration of our mobile intervention involving a cohort of primary care patients with SUDs. We finally discuss implications of our findings for future research and system design, including how to improve model performance, and how classification can serve as the basis for directing attention and resources to those who need them.

### Online Support Forums

SUDs are among the most common mental health disorders in the United States, with over 20 million adults affected in 2013 [5]. SUDs precipitate distress for sufferers and their communities, as well as serious health consequences [17,18]. Although many individuals with SUDs make attempts to stop using substances, resumption of risky substance use, or relapse, is extremely common [19]. With intensive SUDs treatments being time-limited, it is crucial to find ways to extend recovery support to prevent relapse in the long term [20].

Mobile phones and internet use are now ubiquitous in the United States [21], with one consequence being that individuals with mental health challenges can access social support despite physical distance and at any time of day [22]. Often, this support comes from others who share the same mental health concern, as occurs via digital peer-to-peer forums where participants seek help on an “as needed” basis, and provide it to others [23,24]. Such forums typically involve anonymity or pseudo-anonymity, allowing for candid disclosure of personal and stigmatized issues and experiences [25]. Content analyses show that participants in SUDs forums disclose a variety of recovery challenges, prompting exchange of informational and emotional support [26-28].

### The Role of Moderators

Although discussion forums offer a valuable arena for peer-to-peer exchange, moderators can also play a key role. For instance, those in recovery must manage their *own* health issues, limiting the time and energy that can be applied to help others [29]. In addition, although peers can offer first-hand experience related to coping and recovery, they may lack expertise necessary to guide decision making about clinical issues [30]. In contrast, moderators often have knowledge of intervention components and health behavior change processes and may recognize instances where contact or treatment is appropriate [31,32,10]. Moderators may additionally engage in pseudo-therapeutic activities such as offering emotion-focused support or assisting participants in reassessing dysfunctional perspectives [11] and may be more effective than peers in motivating individuals earlier in their behavior change process [33].

The presence of moderators in digital health forums has been associated with benefits. Notably, studies have found greater participation and expressiveness in moderated health forums relative to unmoderated ones [34,35]. In a mobile SUDs intervention for drug court participants, trained staff played a central role in discussion networks, with many participants communicating only with staff [36]. Prior work has also found that staff can enhance the success of digital mental health interventions regardless of formal clinical training [37].

### Machine Learning Applications to Moderator Engagement

To “scale up” digital interventions, designers must take steps to support and streamline moderators’ work. Fortunately, such efforts can make use of extensive data generated as participants engage with technologies. A rapidly growing research area centers on leveraging the digital traces of participants’ activities to gain insights into the changing contexts within which participants are embedded and the psychological states they experience [38,39]. Digital trace data collected through mobile phones may include sensor data (eg, geolocation, accelerometry), as well as patterns of engagement with the intervention itself, and the content of messages exchanged.

By capturing spontaneous, first-hand accounts of authors’ beliefs, feelings, and experiences, text-based messages offer particularly powerful insights into wellness, including the risk of mental health-related outcomes [15]. For instance, prior research has shown that linguistic qualities such as self-focus (as conveyed in pronoun use) can distinguish those who go on to post about suicidal ideation [40], and that negative affective language and swearing can identify individuals who go on to relapse in alcohol recovery [41]. These approaches rely on automated linguistic analysis as described in greater detail below.

Text-based features of user-submitted messages can now be efficiently extracted through a range of computational approaches. One of the most common approaches, BoW, involves representing each message in terms of occurrences of individual words, or “unigrams.” After throwing out extremely common words, and grouping together words with the same stem, a message is represented as a vector formed by the occurrence rate of each stem, relative to that stem’s overall occurrence in the full set of messages. In contrast, dictionary-based approaches search within a message for lists of words corresponding to relevant concepts. For instance, LIWC searches for words representing discussion topics (eg, health, family), psychological dimensions (eg, affect, cognition), and linguistic characteristics (eg, pronouns, conjunctions). LIWC then computes the percent of words in a given message that fall in each category. LIWC has been widely used in research, with studies showing that its categories predict health-related states including suicidality, depression, and dementia [42-44].

Relevant to this study, recent work also uses the above approaches to detect self-disclosure in online forums, defined as messages wherein participants convey personally relevant thoughts, feelings, and experiences [45-47]. In the context of support forums, self-disclosures offer a promising opportunity for intervention (eg, by moderators), because participants are revealing and working through personal issues, and may be actively seeking help [46]. The prior literature suggests that self-disclosure messages involve telltale linguistic cues that aid automatic detection. For example, one study identified a number of LIWC categories predictive of self-disclosure sensitivity, including third person pronouns and discussion of family, sex, death, and negative affect [48]. In another study, individual words conveying affect (eg, “happy,” “love,” and “hate”) were characteristic of mental health–related self-disclosure [49].

Human expertise can also play an important role in guiding the development of language-based models. In supervised machine learning, an expert will designate a subset of messages as belonging to a category of interest (such as mental health risk), and the features of labeled messages are then used to predict whether an unlabeled message would fall in the same category. Labeled data can be generated in a number of ways. For instance, naturally occurring response patterns can be used, such as where Huh and colleagues [12] labeled as problematic those messages to which moderators had previously responded in a health support forum, using their linguistic features to classify new messages that moderators would likely be interested in. Alternately, human judgment can be used to generate each label in the training set, as was implemented in efforts to detect suicidality in an online discussion forum for youth [50,51]. This approach recognizes that moderators’ response patterns do not always clearly follow from the level risk a message indicates. For instance, responding to a message need not represent concern, but could reflect interest in a particular topic or investment in an ongoing relationship. Using SVM, boosted decision trees, and other models, researchers were able to achieve F-scores over 0.9 in identifying messages urgently requiring response [50,51].

### This Study

In online addiction recovery forums, messages can be posted at any time of day or night, and some convey serious or time-sensitive problems. To offer timely help, moderators must continually review new content, but this task is demanding, with potentially dozens or hundreds of new posts to consider every day. Therefore, this study focuses on automatic detection of messages suggesting risk. For instance, interpersonal conflict, legal issues, personal traumas, or encounters with substance use cues could all represent threats to recovery in a substance abuse context [52,53]. Furthermore, these events could inspire psychological states associated with relapse, such as negative affect, cravings, or reduced self-efficacy [54]. Although circumstances and states can be conveyed in a variety of ways, prior literature leads us to anticipate that common language elements should emerge making recovery problems amenable to detection.

This paper contributes to the literature on digital SUDs interventions on several fronts. First, this work has practical application to efficiently capturing concerning content, so that forum moderators can respond in time. Efficient engagement by experts has been identified as a priority for extending the effectiveness of digital mental health interventions [13]. Second, the methodological contribution of our work involves comparing common computational linguistics and machine learning approaches and determining which are suited to the context of mental health risk in support forums.

As far as linguistic analysis, we compare performance of 2 techniques and their hybridization. First, BoW is driven by word-level usage in a given dataset and may therefore have an advantage for recovery-specific words (eg, “drink”). In contrast, our dictionary-based approach, LIWC, characterizes messages along general psychological and linguistic dimensions. Through building on prior knowledge about how words relate to established psychological constructs, LIWC offers potential efficiency, interpretability, and theoretical traction; however, its distinct disadvantage is that its dictionaries are not recovery specific. Thus, although LIWC contains a general category for “health,” it lacks dictionaries corresponding to concepts like “relapse” or “cravings.” Given these trade-offs, it is unclear whether BoW or LIWC will perform best.

Importantly, LIWC and BoW differ in their treatment of common words. The BoW framework retains words that are distinctive of the data at hand. Words with consistently high use across contexts, such as “I” and “we,” are considered insignificant within the BoW framework and typically discarded. In contrast, LIWC computes usage rates of these and other so-called “function” words (eg, pronouns, conjunctions, prepositions), which lack content but hold sentences together [55]. Despite their apparent banality, function words have proved powerful in predicting well-being, with pronouns receiving substantial attention in the mental health domain as a gauge of social integration [40,56,57]. Not surprisingly, personal pronouns also indicate self-disclosure, as they can show that individuals are talking about themselves [49]. In comparing BoW with LIWC and hybrid approaches, we therefore pay particular attention to performance improvements related to function words. In a more general sense, we aim to identify linguistic features most central to manifesting recovery problems, including discussion of substance use triggers, affective states, cognitive processes, and function words.

We also attempt to identify well-performing machine learning approaches. We focus on decision trees with and without boosting, as well as SVM, approaches with good performance in prior social media data [58,59].

Finally, we consider our results in relation to several key features of the domain of recovery support. First, recovery support is an arena where false negatives may be problematic, as missing an opportunity to intervene could allow a problem to escalate, even precipitating relapse. Therefore, in generating gold standard data, we emphasize the importance of establishing a reliable definition of “recovery problems” that is broad enough to capture potentially concerning content. We also reflect our concern about false negatives by prioritizing sensitivity in weighing classifier performance. Second, we seek machine learning methods that can offer insights into the particular language patterns associated with recovery. Decision trees may have an advantage in this regard, as they provide a visualization of the mechanisms of classification that may be helpful to establish face validity among stakeholders [59]. Finally, computational linguistics approaches have different implications for implementing classification in real-time, which we discuss.

## Methods

### Intervention

Data for this study came from a mobile phone–based intervention that provides on-demand services for recovery maintenance and relapse prevention. These services include informational pages, self-management tools (eg, self-help meeting directories, surveys), and peer-to-peer discussion forums. The intervention has been described in detail elsewhere, and it demonstrated efficacy in reducing risky drinking days by more than half relative to a control group [8]. We used data from 2 studies of the system: (1) a clinical trial involving individuals discharged from alcohol treatment (study 1) [8] and (2) an implementation study in primary care, involving individuals who used either alcohol or illicit drugs (study 2) [60]. The institutional review board at the University of Wisconsin-Madison approved both studies. Study participants provided informed consent for collection and use of their data for research (not shared beyond the team). These data included a log of all uses of the intervention and the content of communications exchanged within the intervention.

Study participants were provided with a mobile phone loaded with the intervention: either the Palm Pre with the Palm OS (Palm, Inc, Sunnyvale, CA) or an HTC Evo running Android 4.4 (HTC Corporation, Taiwan). In study 1, 130 participants posted on the forum. They were 56.2% (73/130) male and had a mean age of 38 years (SD 9.7). Participants wrote approximately 20 messages each (average length: 31 words). In study 2227 participants posted on the forum, and they were 53.3% (121/227) male, with a mean age of 42 years (SD 10.7). Participants wrote approximately 69 messages each (average length: 29 words).

This study focuses on text-based messages that were exchanged in the system’s discussion forum, where participants could either start new threads on a topic of their choosing or respond to existing threads. All forum messages were visible to those on study, but study 1 forums were gender segregated. Moreover, 3 members of the research team also monitored the forums (authors GL, FM, and KP). Although the moderators lack clinical background, they are experts in digital health support for self-management of chronic conditions, including addiction recovery.

As mentioned earlier, gold standard data in this study substantially differ from those used in some prior work using moderators’ natural response patterns [12]. We instead developed and applied a standardized, reliable codebook for capturing recovery risk. The first author first conducted an initial interview with the 3 moderators to understand their role in the forum and which messages would be considered worthy of intervention, and then consulted with them throughout the hand-labeling process to ensure our process captured messages of concern.

### Computational Linguistics

We represented discussion forum messages using a BoW model, the LIWC program, and a hybrid approach.

The BoW approach represents each message in a feature space characterized by word counts. Common words were discarded, and remaining words were reduced to their stems using the Lancaster Stemmer from the NLTK stem package in Python and the NLTK word_punct tokenizer. For example, the stem “drink” would capture “drinking,” “drinkin,” “drinker,” “drinks,” and so on. We also wrote an additional filter to remove emoticons and other nonstandard characters. After grouping words according to their stems, Term Frequency-Inverse Document Frequency (TF-IDF) weighting was applied to calculate the occurrence rate of each specific stem in a message, offset by the importance of the stem in the entire corpus. Specifically, TF-IDF for a term is expressed as the term frequency (the number of times a word appears in a document divided by the total number of words in that document) multiplied by inverse document frequency (log of: total number of messages in a corpus divided by the number containing the term), thus adjusting for the fact that some words appear more frequently than others in general [61]. Once computed, the TF-IDF weights are used to form a vector representation of each message. After discarding common words, our BoW representation utilized 4247 unique unigrams as features.

The LIWC 2015 program computes rates of using words that fall within approximately 90 categories representing linguistic characteristics (eg, personal pronouns), topics of discussion (eg, family), affect (eg, anger), and cognitive processes (eg, insight) [16]. Each category corresponds to a predetermined dictionary of related words and word stems. Therefore, each message is represented as a 90-dimensional vector, with each dimension corresponding to a category such as “pronouns” and “positive affect.” The value in each dimension is computed as the number of words from the message belonging to that category divided by the total number of words in the message. For example, “personal pronoun” is one of the features scored by LIWC. In the message “I am doing well,” 1 out of the 4 words are personal pronouns, and so the LIWC score would be 1 out of 4 words or 25%.

In a hybrid approach, we exploit linguistic features from both BoW and LIWC. In other words, for a given message, word frequencies of the most important features from the TF-IDF matrix and the percentages falling in the most important linguistic categories from LIWC are stacked together to form a single feature vector. Given that combining too many features can inhibit performance by introducing noise [62], we utilized a subset of features from each representation. After ranking features according to their importance for a random forest model [63], we picked up to 10% of the most relevant features from BoW and LIWC to form a new feature set. Feature importance is calculated using the Gini Impurity measure, defined as the sum across the number of splits over all trees containing a feature, divided by number of samples in each split [64]. The hybrid approach included 310 features.

### Machine Learning Techniques

With numeric representations of each message in our training set, and a corresponding label (recovery problem or no recovery problem), we trained 3 candidate binary classifiers for our task: SVM, decision trees, and boosted decision trees. SVM is a widely used technique and involves defining an optimal hyperplane to distinguish between items falling in classes of interest [65]. Decision trees involve segmenting the feature space into a number of simple regions [66]. In a series of decision steps, represented as branches, observations are made about an item (eg, the frequency at which a particular word is used within the message), leading to corresponding conclusions about the appropriate class (represented in the leaves). Finally, a related approach, boosted decision trees, involves an ensemble of decision trees where each tree learns by fitting the residual of the trees before it, allowing iterative improvement in performance. Python scikit-learn was used for machine learning [67].

As our datasets feature unbalanced classes (ie, messages including “recovery problems” are outnumbered by messages without them), we compensated for this imbalance by oversampling from the minority class. Specifically, we used the Synthetic Minority Oversampling Technique to generate synthetic samples from the minority class [68]. Rather than creating exact copies, the algorithm samples 2 or more similar instances, with similarity being calculated by a distance measure, (eg, Euclidean, Cosine), and then slightly perturbs these instances to create synthetic samples.

Once our classes were balanced, we trained our classifiers using labeled training data from study 1, the clinical trial for those completing alcohol treatment (n=2581), and calculated parameters for each machine learning model using k-fold cross validation. Next, we tested the best performing models in labeled messages sampled from study 2 (n=800) with its primary care population. We report F-scores, as well as sensitivity (the proportion of correctly identified true positives), specificity (the proportion of correctly identified true negatives), and area under curve (AUC). We also describe example decision trees that illustrate classification logic.

## Results

### Identifying Recovery Problems

Our conversations with moderators first revealed that they recognized a wide range of issues and circumstances as warranting a response (relationship troubles, cravings, etc). Moderators expressed a fear of missing an important message, reporting a preference to have unconcerning messages flagged (false positives) than to miss actual problems (false negatives). Supporting our strategy of hand-labeling problem messages versus using prior response patterns as gold standard data, moderators also reported that contextual considerations influence their likelihood of responding on the forum. For instance, they might be unlikely to respond if participants had already received competent help from peers, or if they had personally had recent contact with participants outside the forum (eg, by phone call or private message). Moderators also stressed that they sometimes miss concerning messages inadvertently.

Guided by this feedback, 3 coders independently reviewed a preliminary set of 200 messages to identify ones they thought disclosed recovery challenges, broadly construed, and then mutually discussed their decisions. Coders arrived at consensus around a rule for coding the entire dataset, when “the writer describes a potential threat to well-being or recovery efforts.” We further specified that the message may express either feeling vulnerable (eg, “I’ve been clean for about 7 months but even now I still feel like maybe I won’t make it”) or may outline a specific incident (eg, “it’s not looking good, they are talking 0 to 5, and that’s not days [in jail]. It’s got my head all f.... up.”). The coding rule also specified that the code should be applied even if the writer conveys that he or she has skills or abilities to handle a given problem (ie, a message may convey both a threat and mastery of that threat at the same time). Thus, by making the coding rule quite general, we avoided some subjectivity involved in making determinations about problems’ seriousness. The first author next overlapped with each other coder on a set of 100 messages, allowing computation of interrater reliability, with average Cohen kappa of .77 for the 2 overlap sets deemed acceptably high [69].

Thus, our codebook captured recovery problems broadly construed. Results of hand-labeling revealed that of the 2581 messages posted to the forum over the course of the study 1, 388 (15%) disclosed some recovery problem. Review of these messages revealed themes including negative affect, cravings, and discouragement. Some described sleep problems, legal issues, medical concerns, unemployment, interpersonal conflict, financial worries, or housing. In a few cases, the writer simply shared that he or she was “struggling” or having a “hard time.” Some messages relayed relapse. In contrast, messages not relaying recovery problems included small talk, affirmations, bonding, reports of doing well or feeling good, or giving support to others.

### Supervised Machine Learning

To choose an optimal classifier and its parameters, we performed 10-fold cross validation on labeled data from study 1, partitioned into 70% training and 30% test datasets. Error metrics used were the average F-scores and AUC scores. Moreover, a total of 3 basic classifiers were considered (1) SVMs with linear and Gaussian kernels, (2) decision trees, and (3) boosted decision trees. Our results indicated that SVM performed worst with improvements in decision trees and best performance in boosted decision trees where we achieved F-scores of 0.88, 0.89, and 0.94 for the BoW, LIWC, and hybrid approaches, respectively. For the decision tree classifiers, we used tree depth of 3 and a minimum of 10 samples per leaf at termination when using the BoW feature space. When using the LIWC feature space, we used the same tree depth but a minimum of 8 samples per leaf at termination. For the hybrid feature space, we used a slightly deeper tree (depth=4) with a minimum of 11 samples per leaf at termination. Boosting utilized an average of 175 estimators across the 3 feature spaces.

Having set parameters, we trained on all data from study 1 and applied all 3 classifiers to test data in study 2. Recall that study 2 contained messages posted by a separate cohort of individuals with substance use disorders (in contrast to study 1 in which all individuals had alcohol abuse issues). F-scores for SVMs, decision trees, and boosted decision trees in test data are provided in Table 1.

**Figure 1:**
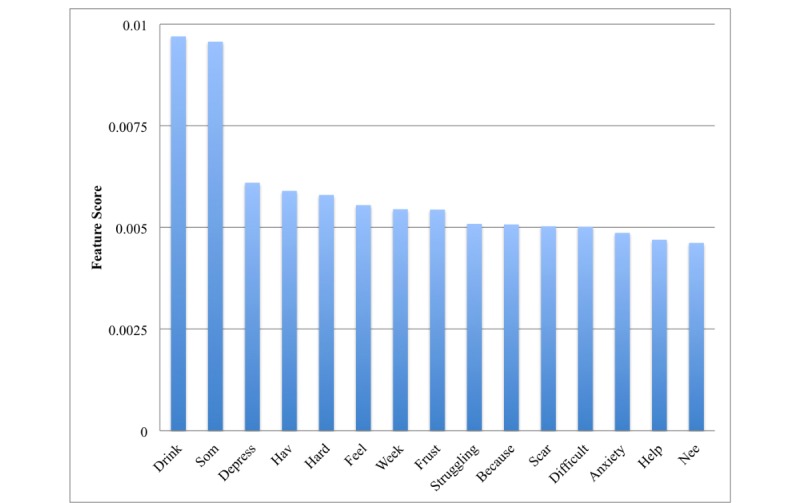
Fifteen most important feature words in the Bag-of-Words (BoW) framework.

**Figure 2:**
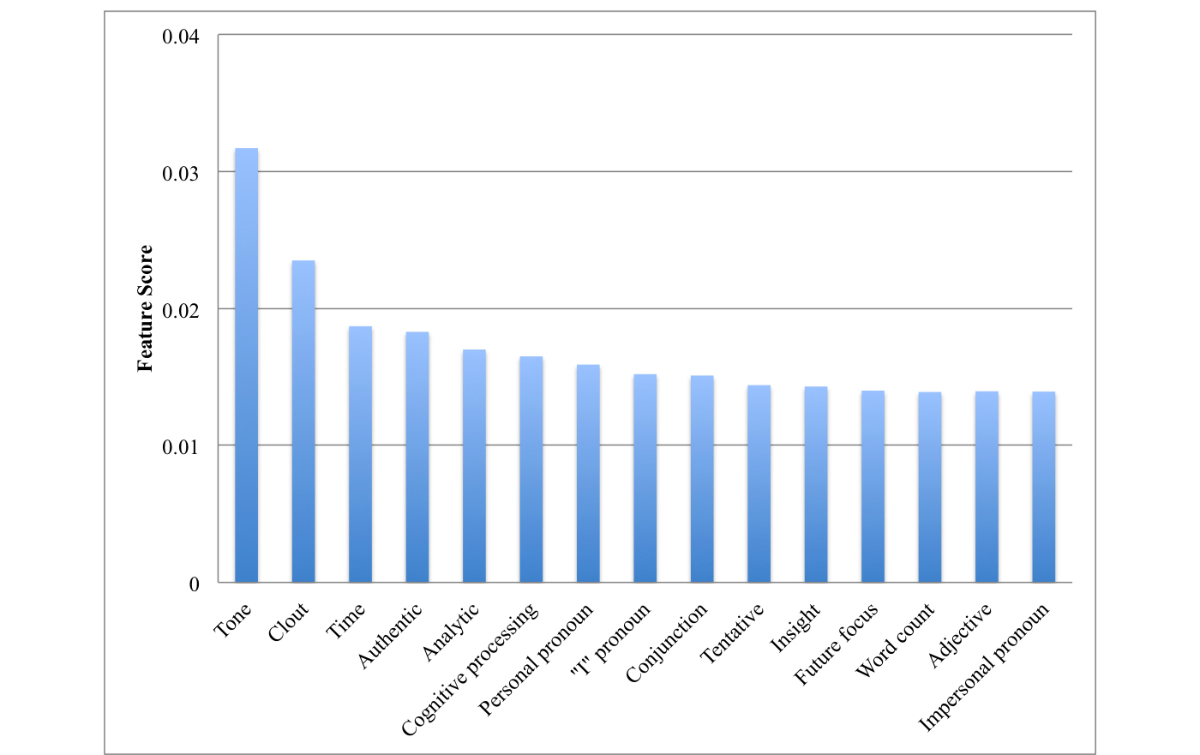
Fifteen most important features in the Linguistic Inquiry and Word Count (LIWC) framework.

Figures 1 and 2 show the top features extracted from the BoW and LIWC representations, respectively. For BoW, top features included words with the stems: drink, som (eg, some), because, hard, depress, feel, and hav (eg, have). For LIWC, top features are tone, clout, time, authenticity, analytic words, and insight words. Moreover, 3 top categories include pronoun forms.

**Table 1:** F-scores reported by 3 classifiers on the test data from study 2.

Classifier	BoW^a^	LIWC^b^	Hybrid
SVM^c^	0.76	0.71	0.76
Decision tree	0.8	0.75	0.77
Boosted decision tree	0.8	0.83	0.85

^a^BoW: Bag-of-Words.

^b^LIWC: Linguistic Inquiry and Word Count.

^c^SVM: support vector machines.

To understand whether demographic characteristics (gender, age) would influence how recovery problems were expressed in language, we conducted additional experiments in the 2581 messages from study 1. In these experiments, we left 1 gender or age out of the training set, reserving this gender or age for a testing set. We used decision tree classifiers with feature representation from LIWC to test this question, finding an F-score=0.76 when training on the 1618 messages posted by women and testing on the messages posted by men, which is identical to cross-validation results achieved with the gender-mixed study 1 sample (F-score=0.76). We used the same approach for age, first leaving out 486 messages from those under 30 years, then 758 messages by those in their 30s, then 881 messages by those posted in their 40s, and finally 309 messages posted by those 50 years or older. The F-scores achieved were 0.77, 0.78, 0.77, and 0.73, respectively. Thus, they were roughly consistent with full study 1 cross-validation, although slightly lower for the 50 years or older group despite the training data being largest.

**Figure 3:**
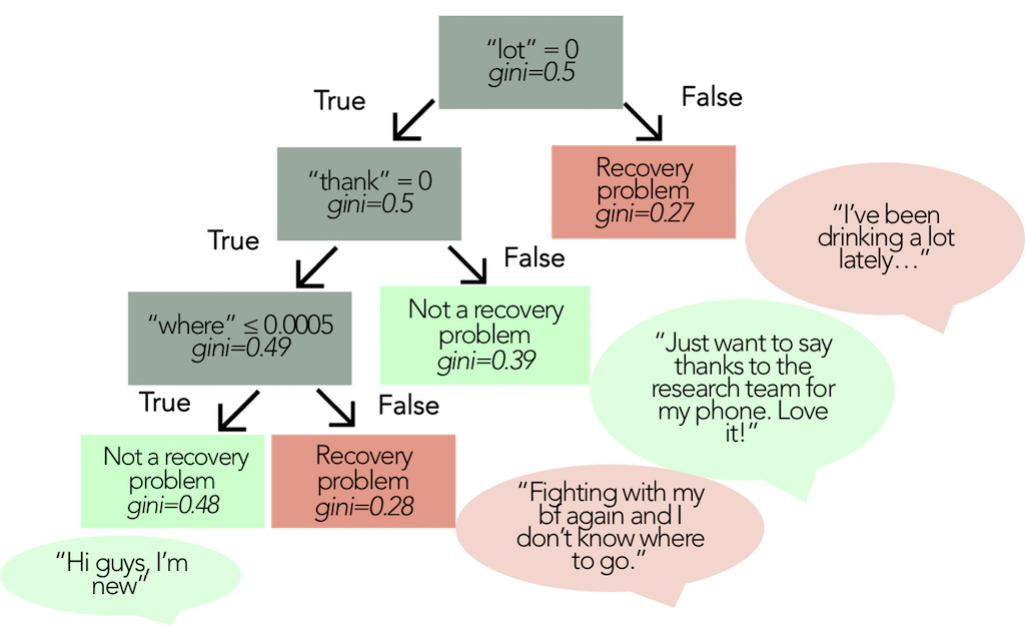
Example decision tree using features from the Bag-of-Word (BoW) approach. Feature importance was calculated using the Gini Impurity measure.

**Figure 4:**
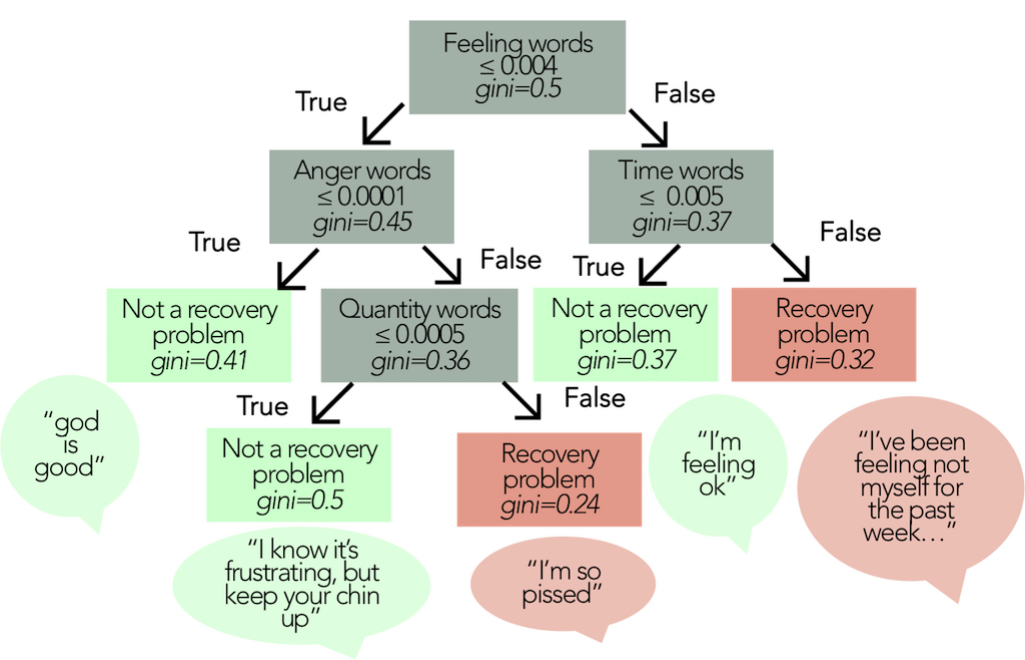
Example decision tree using features from the Linguistic Inquiry and Word Count (LIWC) approach. Feature importance was calculated using the Gini Impurity measure.

We produced decision trees for each approach to represent the relationship between language features in predicting recovery problems. For our models that involved boosting, multiple trees impact each classification decision, so any individual tree will provide only a small window into the logic of classification. Figures 3 and 4 depict truncated exemplar decision trees for the BoW and LIWC approaches. Text in speech bubbles represents messages that would be correctly classified as recovery problem (red) or not a recovery problem (green) by following the associated path. In Figure 3, we can see that the BoW decision tree begins with the stem “lot,” with messages having an absence of the word “lot” (0.0 rate of “lot”) following the “true” branch, and messages with presence of the stem “lot” following the “false” branch and being labeled as “recovery problem” (eg, “I’ve been drinking a lot lately”). For those messages not mentioning “lot,” we next look for the stem “thank,” the presence of which leads to a “no recovery problem” label. For those without “lot” or “thank,” we look for “where,” the presence of which would lead to a “recovery problem” label (eg, “Fighting with my bf again and I don’t know where to go”).

Figure 4 shows the exemplar LIWC tree, which begins with the category of feeling words, producing a categorization of “recovery problem” when paired with time words (eg, “I’ve been feeling not myself for the past week”) but a “no recovery problem” label when mentions of time are below a minimum threshold (eg, “I’m feeling ok.”). For messages without feeling words, the “recovery problem” label would be applied where anger words appear with quantity words (eg, “I’m so pissed!”).

**Figure 5:**
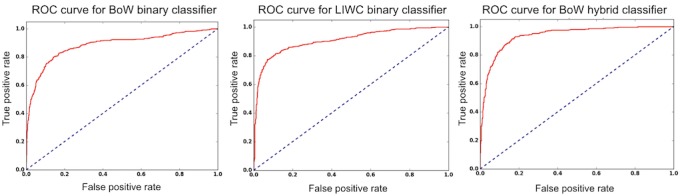
Receiver operating characteristic (ROC) curves for boosted decision tree classifiers on the Bag-of-Words (BoW; left), Linguistic Inquiry and Word Count (LIWC; middle), and hybrid (right) feature spaces.

As boosted decision trees performed better than other classifiers, error analysis was summarized in detail for this classifier, with Table 2 providing specificity, sensitivity, and AUC achieved in the test data for each language processing approach. Results reveal that performance was somewhat improved for hybrid over LIWC and for LIWC over BoW (Table 2). More specifically, the hybrid outperforms the LIWC approach in terms of the F-score and the specificity, but not sensitivity, a point we return to below. The hybrid approach makes for an especially robust classifier as seen from the receiver operating characteristics (ROC) curves in Figure 5. BoW had the lowest sensitivity. For example, the following message was correctly identified by the hybrid and LIWC approaches and missed by BoW: “This is the hardest thing I have ever done. I just wish I felt better bout recovery. I’m nervous I’m gonna go back to my old ways.”

**Table 2:** Error analysis in study 2 for boosted decision trees using 3 language processing approaches.

Language processing approach	Sensitivity	Specificity	AUC^a^
BoW^b^	0.87	0.78	0.85
LIWC^c^	0.91	0.78	0.88
Hybrid	0.88	0.82	0.92

^a^AUC: area under curve.

^b^BoW: Bag-of-Words.

^c^LIWC: Linguistic Inquiry and Word Count.

**Figure 6:**
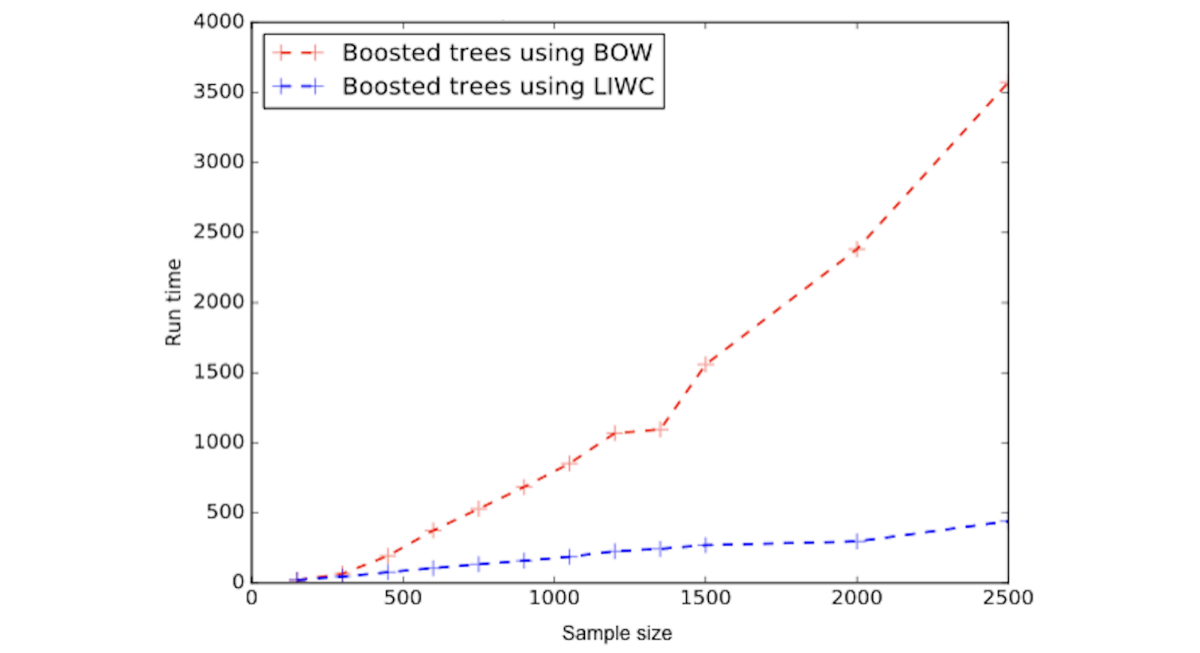
Training times of boosted decision tree classifiers on Bag-of-Words (BoW) and Linguistic Inquiry and Word Count (LIWC) feature spaces.

To understand this classification error, it is helpful to note that the message makes heavy use of personal pronouns such as “I,” which appear in the top 10% of the LIWC features of importance in our datasets but contains just 1 highly ranked unigram from BoW (“hard”). By most metrics, the LIWC and hybrid approach outperform a BoW approach using a boosted decision tree model.

Finally, from Figure 6 we can see the training time for boosted decision trees on LIWC and BoW feature spaces, showing a huge speed advantage for LIWC, a result that was consistent across all classifiers.

## Discussion

### Principal Findings

The burden on mental health services has fueled recent growth of digital interventions, many of which involve text-based forums connecting a network of peers. Forums often operate with assistance from a moderator who steps in as needed, such as when participants’ problems demand more formal intervention or could overwhelm peers’ abilities to help. Although moderators play an important role, their time-consuming work limits the scalability of digital interventions. This study demonstrates a solution that facilitates moderation efficiency while reducing the possibility of overlooking messages of concern: a machine learning-based model to automatically flag messages that disclose recovery problems. For this work, we used several machine learning approaches, with boosted decision trees performing best, while also offering a view into the logic of classification that may be helpful in establishing face validity.

We also represented our data through a number of computational linguistics techniques. Although the BoW approach captured domain-specific language, it performed somewhat worse than LIWC, a dictionary-based approach capturing psycholinguistic features. LIWC may do well in this context because recovery problems have important affective dimensions; prior literature shows that LIWC may perform well in cases where affect is a dominant theme [42]. We further found that a hybrid approach, leveraging a combination of features from the dictionary-based LIWC program and BoW, performed best for classifying our test data with regard to AUC and F-score. However, these improvements were only marginally improved over LIWC alone. LIWC achieved a similar F-score of 0.83 (compared with 0.85 for the hybrid) and actually had a higher sensitivity of 0.91.

Ideally, analysts often seek solutions that maximize performance as measured by the F-score, which in this case points toward the hybrid approach. However, there are times when an analyst might prefer greater sensitivity (avoiding false negatives) over improved specificity (avoiding false positives), including perhaps the context of addiction treatment and other health contexts where missing problematic messages could be costly. Given our desire for high sensitivity, LIWC may even be a preferable option over the hybrid. To put this in practical terms, LIWC correctly classified 116 out of 127 true positives in our study 2 test data, compared with 112 classified by the hybrid approach. These additional 4 messages came at the expense of an additional 29 false negatives. However, given the potential consequences of a missed true positive, the additional review time may be seen as worthwhile, especially in early stages of implementing this sort of classifier when concerns about missing actionable messages may limit adoption. Digital mental health interventions seek to ease burden on providers while delivering care to patients, but adoption requires faith in system performance among those on the “front lines.”

LIWC may also be preferable given its easier real-time implementation. Our experiments showed that LIWC features enabled faster training than BoW. LIWC may also have an implementation advantage as the BoW approach involves calculating TF-IDF scores that reflect the occurrence rate of the word in a single document as well as that word’s occurrence across all documents in a sample. This suggests that BoW may present computational challenges when applied in a “live” forum, as the overall occurrence for unigrams may change as new messages are posted and as new unigrams may emerge over time. On the other hand, as the LIWC dictionaries are broad and fixed, classifiers may work well in a system where messages are continually added.

We found that our classifiers were flexible enough to capture numerous circumstances that present problems in recovery, including interpersonal conflicts, job and housing instability, feelings of hopelessness, and encountering triggers. Despite the variety of problems described, classifiers relied heavily on particular ways of talking about drinking, affect, and context, as evident from the important features extracted for each method. Decision rules using the BoW approach were sometimes based on weights of words explicitly linked to drinking (“drink,” “relapse,” “sobriety,” and so on), but decision trees also revealed the use of certain location and context-related terms (“stay” and “where”) in decision rules. Decision rules from the LIWC approach were rarely based on explicit topics of discussion, but instead reflected characteristics such as tone, affect, insight, and presence of quantifiers and time references, as well as pronouns. In a tree-based approach, it is not simply using words within these categories that matters but couse with words in other categories.

### Comparison With Prior Work

Notably, in all cases, we achieved good performance relative to Huh and colleagues [12], who also attempted to detect appropriate messages for moderator intervention, and who achieved F-scores up to 0.54. This may in part reflect the difference in machine learning approach, as we used boosted decision trees rather than the Naive Bayes technique they report. The improvement may also reflect the labeling process for training data. Specifically, their training approach labeled messages according to whether they actually received a moderator response, presuming these to be messages of greatest concern, but we implemented a reliable human coding process that we thought would minimize error, as moderators’ responses are actually driven by a number of factors beyond the level of concern a message produces.

Indeed, our results are more closely in line with studies that have used hand-labeled data for training. F-scores for our hybrid model are comparable with the best results achieved in a shared task challenge to flag messages for elevated suicide risk in a forum for Australian youth [50] and slightly lower than a follow-up study from the same forum that utilized an ensemble of feature extraction approaches (LIWC, topic modeling, meta-data, etc) [51]. However, it is important to note our more conservative approach of testing our model in a separate iteration of the forum with a separate patient population. Like Conan et al [51], we also obtained better results for boosted decision trees relative to SVM.

### Implications for System Design

Moderators can play a pivotal role in digital forums for at-risk populations but face difficulties keeping up with new content. Recently, scholars have called for improving digital health interventions by emphasizing *efficiency* of human support: the level of increased engagement and intervention effectiveness relative to the effort expended by staff [13]. Our findings demonstrate an opportunity to improve efficiency through automatically identifying, in real time, when participants disclose pressing concerns. Resulting classifications could be easily used to populate an interface to display high-priority content to moderators (see Multimedia Appendix 1 for an example of how our classifier has been applied in our live mobile-based recovery support platform). The interface may also provide moderators with an opportunity to dispute message classifications they view as erroneous, generating data to refine classifiers in the future (See Multimedia Appendix 2). Upon review of flagged messages, moderators might choose to intervene in a number of ways, such as through providing emotional support, directing participants to intervention elements that might suit their needs, or connecting participants with mentors or services.

Although our present solution requires human review and response, it is worth noting an alternate approach of fully automating responses. For instance, flagged messages could prompt the system to provide immediate contact information for treatment providers or emergency services, thus offering support even late at night and early in the morning. Some systems have also used machine learning methods to match newly posted content to semantically similar earlier content, displaying these older messages alongside the responses they generated in case they are useful to the current poster [70]. In addition, more complex dialogue systems have been applied to further reduce the human labor behind digital health interventions, including interactive “conversational agents,” software programs that mimic human conversation, and that may further display human-like cues through voice or visual representations [71,72]. Such techniques are promising but involve trade-offs relative to trained staff who develop personal relationships with participants and can exercise expert judgment [73]. For instance, unlike software programs, moderators can choose to ignore messages they believe are “false positives,” not warranting their expression of concern. Of course, moderators also vary in personal and professional qualities that make them effective. For instance, staff may be particularly successful through conveying a combination of trustworthiness, benevolence, and expertise [74].

In the future, efficient just-in-time support may involve judicious use of both human support and automated messages. Short of full automation, efficiency could be enhanced through providing moderators with a drop-down list of common responses that may be appropriate after a problem is disclosed, with an editor allowing optional personalization. Information about a given participant (eg, risk score from the last completed survey) could also indicate whether a flagged message should be sent to the moderator for a personalized response or managed through automation.

### Future Research Directions

Findings from this study suggest promising areas for future research. First, a number of additional optimizations of our classifiers may be possible. For instance, additional dictionaries have also been developed in the realm of electronic medical records and these could prove promising in capturing recovery-related concepts [75]. Conditional Random Fields methods also work well in classifying natural language [76]. In future, we may also improve our BoW-based model through attention to dimensionality reduction, latent semantic analysis, and potentially extracting bigrams (or trigrams, etc) in addition to unigrams. As far as our hybrid approach is concerned, we might further optimize performance by giving further consideration to the number of features pulled from each component method. Specifically, to determine the number of important features from LIWC and BoW to include in the hybrid model, we tested cut-off points at 5% intervals (10%, 15%, 20%, etc) and found the best results for 10%, but more fine-grained adjustments could be tested, including plotting F-score relative to the number of features, and perhaps allowing for different cut-offs for LIWC and BoW.

Although our models were robust regardless of type of substance of abuse (which varied across Studies 1 and 2) and by gender, our leave-one-out experiments suggest that further research may also be needed to understand if older adults use similar language to convey recovery problems. We also did not test our model across differences such as race or education, leaving it unclear whether our models would work well in populations of different compositions.

Models might also take additional data into account. This analysis was conducted at the message level, but it may be possible to improve our models by considering each individual’s pattern of messaging. Those who habitually post recovery problems may require a different level and style of response than those who escalate posting of worrisome messages. Other system use or sensor data may also inform our model, such that patterns of reading messages, interacting with intervention features (eg, pressing a “panic button”), or moving to new geographic locations may be integrated into decision rules around moderator involvement [77]. Similar work in the domain of suicide risk has incorporated additional features reflecting metadata from the discussion forum (eg, How many usernames are referenced in a message? Where does a message fall in sequence within a thread?) [51].

Ultimately, the efficiency of our approach to flagging concerning messages should be addressed empirically, such as through a trial randomizing some participants to a system where moderators manually review the forum and others to a system where moderators rely on text-based classification. Outcomes may include moderators’ workload as well as patients’ satisfaction and health outcomes. Further research is also needed to establish how to best intervene after a recovery problem message, including through personalized responses from moderators or automated messages.

A final future direction relates to privacy. Our surveillance approach offers opportunities to intervene early to help those in need, but introduces an important trade-off as far as privacy. Specifically, we use passively collected data to infer underlying risk levels that patients may not even be aware of, with these data being highly sensitive [50]. Future research is needed to clarify how patients understand uses of their data for surveillance, how they balance surveillance and privacy concerns, and the contexts under which they find surveillance acceptable. In this study, it is possible that we allayed some privacy concerns by recruiting patients through trusted treatment providers and clinicians and obtaining informed consent, but patients may have greater privacy concerns in the domain of commercial mental health platforms.

### Limitations

This study has limitations. First, our approach would not allow us to assist participants who do not post on a discussion forum. Furthermore, we do not look at private messages, where participants potentially disclose even more sensitive information [50]. In addition, as we did not label subtypes of recovery problems, it is possible that our classifier may be biased toward recognizing certain types of common problematic messages over others. Future work should consider coding subtypes of recovery problems. For instance, relatively rare problems that are nonetheless highly concerning may include mentions of suicide risk or solicitations to buy or sell drugs. Finally, one of the core strengths of our dataset is also tied to one of our study limitations. Specifically, we have access to a dataset of anonymous messages exchanged in a system restricted to those who share a SUDs diagnosis (a condition of study eligibility). These factors mean that discussion may be particularly candid and may offer unusual insight into mental health risk. At the same time, these considerations imply that existing labeled datasets cannot easily be adapted to train classifiers within our dataset. Our model leverages a relatively small set of training messages, which has implications for the machine learning approaches available and the results obtained.

### Conclusions

Digital interventions hold promise to offer cost-effective, constantly available support to those in recovery, and to reduce human workload relative to face- to-face SUDs interventions. However, human support still plays a vital role in many effective digital interventions. For interventions involving discussion forums, trained moderators can respond in real time to help participants who are facing challenges. Yet, these moderators must dedicate substantial time and effort to manually review newly posted messages to identify serious problems, and the process can be error-prone. Our results show that message content can be effectively leveraged toward facilitating just-in-time supportive intervention. Language-based classification models have potential for massive scalability as digital interventions for addiction support continue to expand.

Individuals’ language use, both through its content and composition, offers a means of understanding psychological states and traits. Our work expands on the existing literature by combining and layering computational linguistics and machine learning techniques in the context of streamlining human support within digital substance abuse recovery interventions. Yet, this work also has theoretical and methodological value beyond this specific context, suggesting useful directions for applying language classification to digital mental health more broadly.

## Appendix

Multimedia Appendix 1 Interface for moderator to review messages classified as indicating recovery problems.

Multimedia Appendix 2 Interface for moderator to provide feedback on message classification.

## Abbreviations

AUC: area under curve
BoW: Bag-of-Words
LIWC: Linguistic Inquiry and Word Count
ROC: receiver operating characteristics
SUD: substance use disorder
SVM: support vector machines
TF-IDF: Term Frequency-Inverse Document Frequency
