# Supplementary material for: Detecting Recovery Problems Just in Time: Application of Automated Linguistic Analysis and Supervised Machine Learning to an Online Substance Abuse Forum
Source: J Med Internet Res. 2018 Jun 12;20(6):e10136. doi: 10.2196/10136 (PMC6019846; doi:10.2196/10136)
Supplement: Multimedia Appendix 1 [file jmir_v20i6e10136_app1.pdf]

A-CHESS Administration

Logout

« Hide Sidebar

Home

Administration

- Agencies
- Users
- Counselor Dashboard
- Moderator Dashboard

Discussions

- Flagged Messages 91
- Groups

Content Management

- Documents
- Events

User Data

- Use Data Report
- Recovery Motivations
- Weekly Surveys

Tools

- Tech Support Tools
- HCV / HIV Stages

Flagged Messages

Export Moderation Data

☐ **Show messages seen by a moderator**

Sort by: Date Posted | Date Flagged | Author

Message Thread

Clean from the dope but...

So I've been clean from dope for 3 months now, however, I suffer from depression and lately it's been getting alot worse. My doctor put me on Zoloft for a while but I didn't like it for other reasons and it didn't help much. I had some cocaine and realized, obviously, it helped numb my thoughts. I know this is terrible but it's the only thing that helps me so I've been getting it all the time. Growing up and through 5 years in the Marine Corps, I never dealt with depression or anxiety and when I was on dope and had a problem I would just hit up my dude and forget about things. I think I'm substituting one for the other. When I have nothing, I won't be able to sleep and I'll sneak away from the wife and kids in the middle of the night and cry uncontrollably like a baby mainly because how ashamed I am of how my life has turned out due to my poor choices. And those choices haven't only affected me. And then there is a billion other things running through my head! Sometimes it gets so bad I feel like I'm gonna pass out because I can't breath while I'm freaking out crying. But when I have the coke I don't think about any of that shit and I just go with the flow. I think I have permanently ruined the man I once was! I need help!

Posted by on Jun 20, 2017 at 2:38 PM
Flagged Jun 22, 2017 at 8:00 AM

Moderate | Go to thread

Message Thread

Clean, but wild thoughts.

I've been clean for 9 months and counting and I love my life clean but why do i think about using even when I know how much I will lose? I have my 8 month old son and just so amazingly happy. I don't think I'd ever use again but shut don I think about it? I hate it! This is my 4th time with recovery time but this time feels like the winner (: I am on 12mg of suboxone too

Posted by D on Jul 1, 2017 at 5:21 PM
Flagged Jul 1, 2017 at 6:00 PM

Moderate | Go to thread
