# Supplementary material for: Detecting Recovery Problems Just in Time: Application of Automated Linguistic Analysis and Supervised Machine Learning to an Online Substance Abuse Forum
Source: J Med Internet Res. 2018 Jun 12;20(6):e10136. doi: 10.2196/10136 (PMC6019846; doi:10.2196/10136)
Supplement: Multimedia Appendix 2 [file jmir_v20i6e10136_app2.pdf]

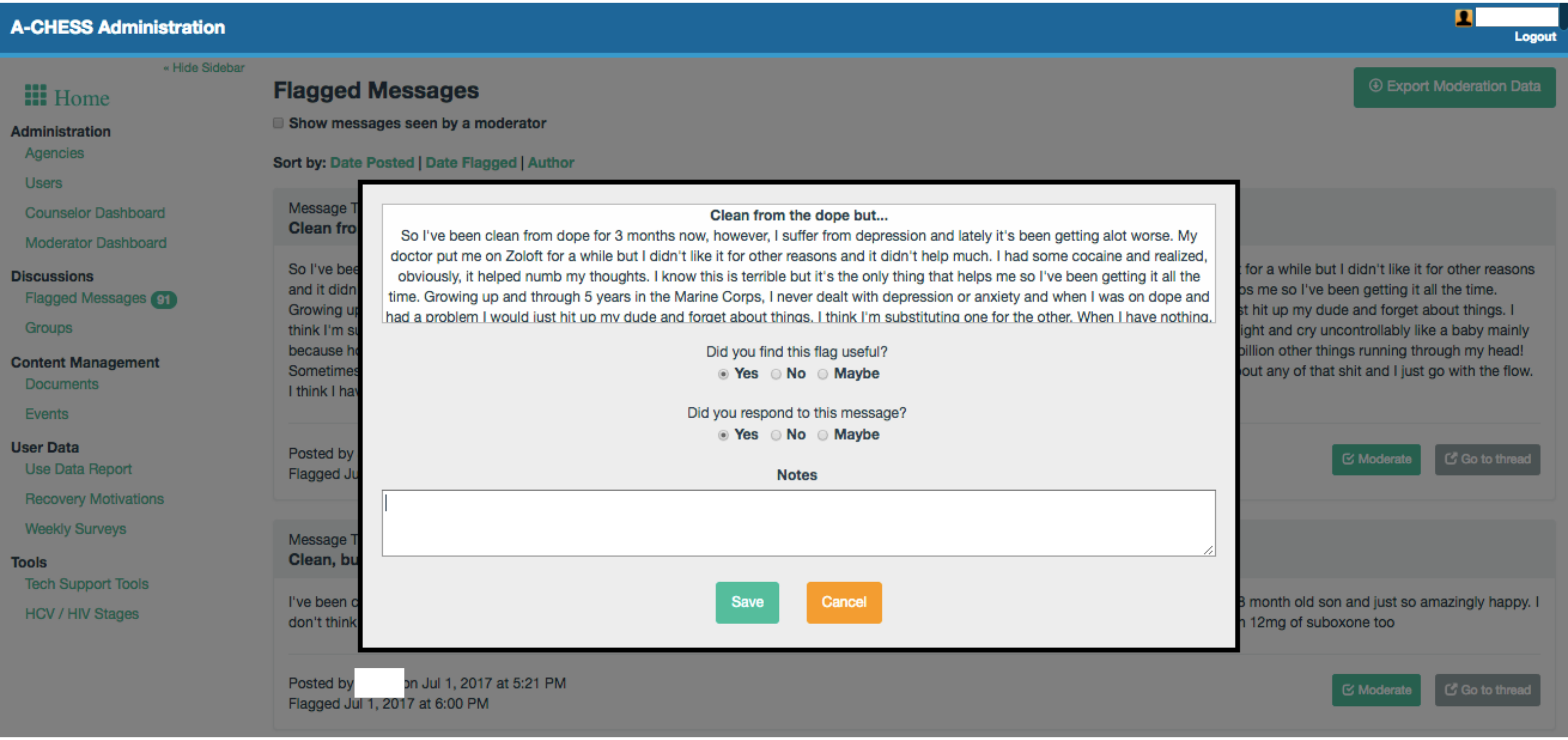
A-CHESS Administration
Logout
« Hide Sidebar
Home
Administration
Agencies
Users
Counselor Dashboard
Moderator Dashboard
Discussions
Flagged Messages 91
Groups
Content Management
Documents
Events
User Data
Use Data Report
Recovery Motivations
Weekly Surveys
Tools
Tech Support Tools
HCV / HIV Stages
Flagged Messages
Export Moderation Data
Show messages seen by a moderator
Sort by: Date Posted | Date Flagged | Author
Clean from the dope but...
So I've been clean from dope for 3 months now, however, I suffer from depression and lately it's been getting alot worse. My doctor put me on Zoloft for a while but I didn't like it for other reasons and it didn't help much. I had some cocaine and realized, obviously, it helped numb my thoughts. I know this is terrible but it's the only thing that helps me so I've been getting it all the time. Growing up and through 5 years in the Marine Corps, I never dealt with depression or anxiety and when I was on dope and had a problem I would just hit up my dude and forget about things. I think I'm substituting one for the other. When I have nothing,
Did you find this flag useful?
Yes No Maybe
Did you respond to this message?
Yes No Maybe
Notes
Save
Cancel
Moderate
Go to thread
Posted by
n Jul 1, 2017 at 5:21 PM
Flagged Jul 1, 2017 at 6:00 PM
Moderate
Go to thread
